# Strand-Specific RNA-Seq Reveals Ordered Patterns of Sense and Antisense Transcription in *Bacillus anthracis*

**DOI:** 10.1371/journal.pone.0043350

**Published:** 2012-08-22

**Authors:** Karla D. Passalacqua, Anjana Varadarajan, Charlotte Weist, Brian D. Ondov, Benjamin Byrd, Timothy D. Read, Nicholas H. Bergman

**Affiliations:** 1 School of Biology, Georgia Institute of Technology, Atlanta, Georgia, United States of America; 2 National Biodefense Analysis and Countermeasures Center, Frederick, Maryland, United States of America; 3 Division of Infectious Diseases & Department of Human Genetics, Emory University School of Medicine, Atlanta, Georgia, United States of America; Duke-National University of Singapore Graduate Medical School, Singapore

## Abstract

**Background:**

Although genome-wide transcriptional analysis has been used for many years to study bacterial gene expression, many aspects of the bacterial transcriptome remain undefined. One example is antisense transcription, which has been observed in a number of bacteria, though the function of antisense transcripts, and their distribution across the bacterial genome, is still unclear.

**Methodology/Principal Findings:**

Single-stranded RNA-seq results revealed a widespread and non-random pattern of antisense transcription covering more than two thirds of the *B. anthracis* genome. Our analysis revealed a variety of antisense structural patterns, suggesting multiple mechanisms of antisense transcription. The data revealed several instances of sense and antisense expression changes in different growth conditions, suggesting that antisense transcription may play a role in the ways in which *B. anthracis* responds to its environment. Significantly, genome-wide antisense expression occurred at consistently higher levels on the lagging strand, while the leading strand showed very little antisense activity. Intrasample gene expression comparisons revealed a gene dosage effect in all growth conditions, where genes farthest from the origin showed the lowest overall range of expression for both sense and antisense directed transcription. Additionally, transcription from both strands was verified using a novel strand-specific assay. The variety of structural patterns we observed in antisense transcription suggests multiple mechanisms for this phenomenon, suggesting that some antisense transcription may play a role in regulating the expression of key genes, while some may be due to chromosome replication dynamics and transcriptional noise.

**Conclusions/Significance:**

Although the variety of structural patterns we observed in antisense transcription suggest multiple mechanisms for antisense expression, our data also clearly indicate that antisense transcription may play a genome-wide role in regulating the expression of key genes in *Bacillus* species. This study illustrates the surprising complexity of prokaryotic RNA abundance for both strands of a bacterial chromosome.

## Introduction

The RNA-seq approach is an unbiased sequencing-based method for characterizing RNA that has greatly enhanced our ability to view the transcriptomes of both eukaryotes [1], [2], [3] and prokaryotes [4], [5], [6]. Previous hybridization-based methods for exploring gene expression, such as those based on microarray technology, were limited to intersample comparisons. However, the unbiased and quantitative nature of RNA-seq allows a more absolute measure of RNA abundance, and because it captures the sequence as well as abundance of each RNA, it can reveal aspects of transcriptome structure on a genome-wide scale, such as operons for prokaryotes, splice variants in eukaryotes, and transcriptional activity within non-coding regions such as riboswitches, small non-coding RNAs, and intergenic and untranslated regions.

In recent years, transcription and RNA function have been shown to be more complex and varied than expected. One surprising finding has been the observation of widespread antisense transcription in both eukaryotes and prokaryotes [7], [8], [9]. However, the precise function and mechanism(s) of this seemingly ubiquitous phenomenon are still unknown, particularly in bacteria, though it seems likely that antisense RNA often plays a role in regulating gene expression [10]. It is generally believed that within the bacterial cell, the presence of a complementary antisense copy of RNA will hybridize to the normal, sense copy of mRNA, causing it to be degraded or translated less efficiently [11]. However, regulating gene expression in this way could be metabolically more costly to the cell due to the use of energy and metabolites.

Although several studies have shown that antisense transcription may be widespread in bacteria [5], [9], [25], and several high-resolution bacterial transcriptomes have been reported [12], [13], a global strand-specific quantification focusing specifically on the frequency distribution of antisense transcripts has not been reported. Here, we describe a detailed genome-wide analysis of transcription in the Sterne 34F_2_ strain of the bacterium *Bacillus anthracis*, the causative agent of anthrax [14], [15]. Sterne is an attenuated strain of *B. anthracis* harboring one of two virulence plasmids (pXO1), and has served as a model for the general bacterial physiology of more fully virulent strains [16], [17], [18]. Gene expression in *B. anthracis* has been examined extensively [19], [20], [21], [22], [23], and thus, this study expands on previous work by using strand-specific RNA-seq to explore both sense and antisense transcription in *B. anthracis* populations from four different growth conditions. We observed that transcription is heavily dependent on genome architecture, such that antisense activity was overrepresented on the lagging strand, where RNA transcription and DNA replication occur in opposing directions, while sense-directed transcription was only slightly more prevalent on the leading strand. Additionally, the highest levels of sense transcription were mainly on the high-copy regions of the leading strand, closer to the origin of replication, indicating possible gene dosage effects. Briefly, gene dosage effects are those caused by the presence of more copies of chromosomal DNA closer to the origin being present during DNA replication, thus providing more template copies of those genes for transcription.

Lastly, our data showed specific examples of unique forms of antisense transcription that both remained constant and changed between bacterial growth conditions. For instance, we observed: (i) abundant antisense within an important sigma factor (*sigA*) in all conditions; (ii) several genes where antisense transcription was much higher than sense directed transcription; (iii) a significant reversal in antisense transcript abundance for two spore-related genes between exponential and stationary phase growth; and (iv) a sharp rise in antisense transcription within several metabolic genes during osmotic and cold stress. Significantly, we verified a subset of our findings using a novel strand-specific assay. Taken as a whole, our data suggest that genome architecture and perhaps species-specific gene content may be important factors in determining the distribution and abundance of antisense transcription in this Firmucute bacterium, where the genomes for this phylum have a high coding strand bias [24].

## Results

### Analysis of Sense and Antisense Transcription in *B. anthracis*

**Figure 1:**
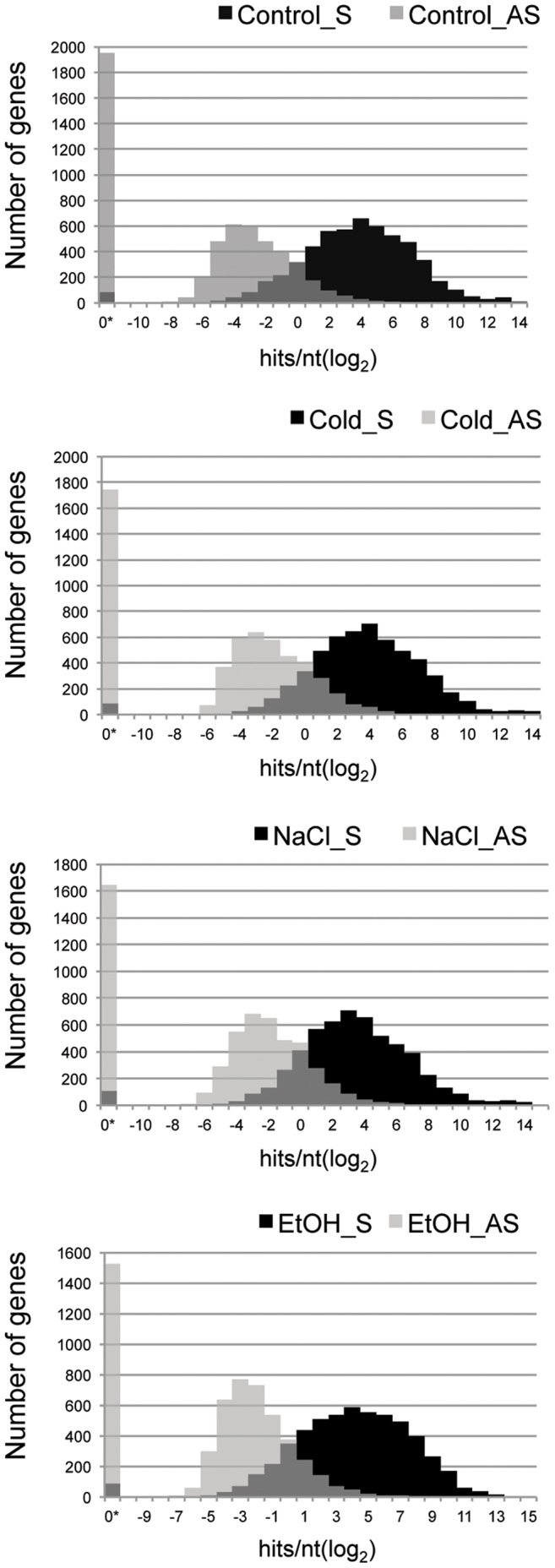
Frequency distributions for Sense and Antisense signals in four unique transcriptome samples. Plots represent the numbers of genes in each range of scores for both Sense and Antisense signals (x-axis = log2 of scores versus y-axis = number of genes within each range). Control = exponential growth in rich medium; Cold = 10 minutes at 17°C; EtOH = 10 minutes at 6% Ethanol; and NaCl = 10 minutes in 0.7 M sodium chloride. 0* = score of 0.00.

**Figure 2:**
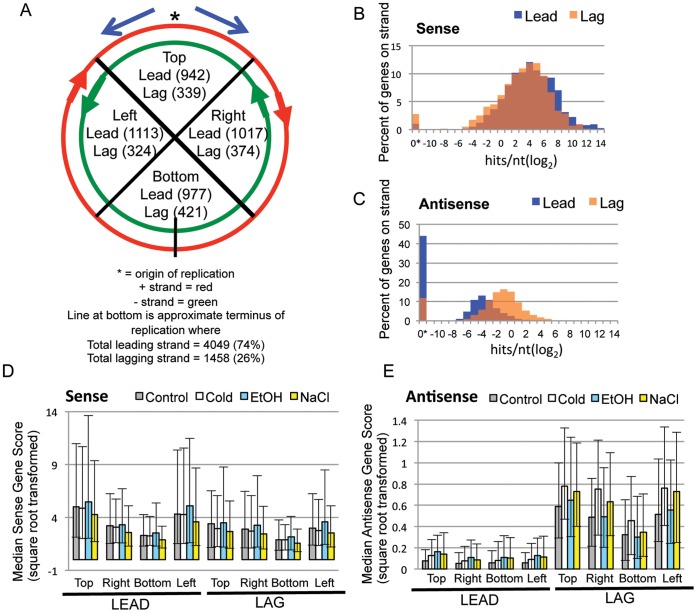
The *B. anthracis* chromosome, Leading and Lagging strand gene score distributions, frequency distributions and median gene expression scores for Sense and Antisense expression by leading and lagging strands. (A) Basic outline of the ∼5.2 Mb chromosome of *B. anthracis*, showing approximate origin and terminus of replication, and the division scheme of genes on leading and lagging strands. Note that red is the plus strand and green is the minus strand, but “leading” and “lagging” directionality switches at the terminus, with “leading” strand genes going in the same 5′-3′ direction of replication, and “lagging” going in opposite direction. (B-C) Frequency distributions of percentage of genes per leading and lagging strands for sense (B) and antisense (C) transcripts in Control sample. (0* means score of 0.00). (D-E) Median gene expression and interquartile range per quadrant divisions outlined in (A) for sense (D) and antisense (E) gene expression.

We used strand-specific RNA-seq (RNA-seq) [6] to define the transcriptome of *Bacillus anthracis* under four unique growth conditions in order to better understand the extent to which antisense transcription is present in logarithmically growing bacteria in different growth environments. We collected RNA from four biological replicates of *B. anthracis* grown to mid-exponential phase in rich medium with no treatment as well as with 10 minutes exposure to 6% ethanol (EtOH), cold stress (Cold), and 0.7 M NaCl (16 samples total - see Methods) [25]. Our goal was to determine the extent to which antisense RNA is produced during bacterial growth under unique growth environments, and whether discrete patterns of antisense transcription could be revealed [4]. The experiments described here represent an extremely high-resolution view of a bacterial transcriptome (22,276 Mb total sequence data generated) (Table S1) using the Applied Biosystems SOLiD next generation sequencing technology. Briefly, sequence reads were mapped to the *B. anthracis* genome, and within each dataset, the coverage depth for each nucleotide in the genome was calculated (i.e., each nucleotide was counted). Each strand was considered independently, such that sense and antisense transcription were measured separately. From these coverage calculations, gene scores (“gene score” = Σ reads per nucleotide/length of gene) were calculated for each of the 5,507 annotated open reading frames encoded within the *B. anthracis* Ames Ancestor chromosome [26], [27] from both the sense and antisense strands, resulting in two unique expression scores per gene - one from the sense strand and one from the antisense (AS) strand. Each gene’s score was calculated independently for each of the 16 samples. Because gene scores are calculated using gene length as the denominator, scores are by definition corrected for differences in gene size, and so can be directly compared. Spearman rank and Pearson unranked correlations measured a high degree of relatedness between biological replicates (r>0.8) at the nucleotide level and no correlation between plus and minus strand measurements within the same sample (Table S2). The high level of correlation between biological replicates showed that the single stranded RNA-seq strategy is extremely repeatable, consistent with the increasing use of this method [28] (see table S3 for all individual gene scores, standard deviations and 95% confidence intervals). After confirming correlation between biological replicates, gene scores for each replicate were normalized based on the total number of unambiguously mapped reads for each sample to correct for differing depths of sequencing coverage. The gene scores for the four biological replicates of each experiment were then square-root transformed for subsequent expression analyses (in order to include gene expression scores of 0 when comparing samples) (Table S3), and also log base 2 transformed (for plotting frequencies in Figures 1 and 2).

Figure 1 shows frequency distributions of gene scores (normalized log_2_ transformed) for each RNA pool with the number of sense-directed gene score frequencies (sense “S”) in black and antisense score frequencies (antisense “AS”) in grey, for all genes. As in a previous RNA-seq study, the log_2_ distribution of transcript abundance followed a smooth, continuous curve [4]. The majority of sense-directed transcripts ranged between 2^2^ and 2^6^ log_2_ hits/nt (for pXO1 plasmid, see Figure S1). Interestingly, the distribution of AS gene scores also followed a continuous distribution, but was shifted to the left of the sense-directed gene scores, showing that overall, AS signals across protein coding genes are at much lower abundance than their sense counterparts (range of log_2_ scores ∼2^−7^–2^4^). As in previous work, the high resolution of RNA-seq revealed that almost all of the genes on the chromosome were represented as sense transcripts (∼98% of genes detected>0.00) [4]. However, for many genes (∼30% for all 4 samples) there was no detectable AS signal (AS = 0.00), suggesting that AS is not random noise distributed evenly around the chromosome.

Before exploring the possible biological significance of AS transcription, we sought to verify that our findings were not a result of mismapping of sequence reads by our mapping algorithm. Therefore, we performed an experiment to assess mapping accuracy for SOLiD reads across the *B. anthracis* genome. We generated 200 50-color strings beginning at every position of the *B. anthracis* genome, with color errors included at random positions to allow for related sequence strings to potentially misalign (see Methods). We then mapped the>10^9^ generated reads using the mapping software SOCS [29] and found that 1,032,172,600 reads correctly mapped, and only 36,800 (0.003%) incorrectly mapped, suggesting that our observation of widespread AS transcription was not due to bioinformatics error.

The simulation results indicated that the range of sense and AS signals were very likely representative of true RNA signals, and so we quantitatively confirmed a representative subset of these genes using the NanoString® nCounter technologies (http://www.nanostring.com) with the original total RNA extractions. The NanoString assay is a very sensitive, hybridization-based assay that measures RNA abundance in a multiplex format using color-coded single-stranded probes (see [30], [31] for an excellent description of the assay). Thus, we selected a set of sense and AS probes for 17 genes that showed a variety of levels of sense and AS transcription in the RNA-seq measurements.

Table 1 shows a side-by-side comparison of the sense and AS gene scores and ratios from RNA-seq and NanoString measurements (Control sample 5 of 17 genes - all data are located in Tables S4 and S5). Note that NanoString nCounter is a fluorescence-based assay, and normalization was done as explained in Methods – thus ratios are results of independent measurements of the RNA as transcript counts (RNA-seq using ribosome-depleted RNA) and fluorescence measurements (NanoString nCounter using pre-ribosome depleted RNA). Replicates were averaged and Spearman Rank correlations comparing RNA-seq and nCounter sense:AS ratios showed that the two methods were highly correlated (note that sense measurements do not correlate to AS measurements – not shown). Taken together with the bioinformatics experiment discussed above, these data strongly confirm the sense and AS signals observed in our single-stranded RNA-seq experiment.

**Table 1:** Strand-specific expression measurements of 5 genes by both ssRNAseq and nanoString Technology^1^ (5 out of 17 represented – all nanoString data in Tables S4 and S5).

Gene name	locus_tag	2Sense ssRNAseq	2AS ssRNAseq	3Sense nanoString	3AS nanoString	4S:AS Ratio ssRNAseq	4S:AS Ratio nanoString
spore germination protein *gerD*	GBAA0148	0.22	27.91	71	943	0.01	0.08
3-deoxy-7-phosphoheptulonate synthase	GBAA2958	600.93	0.04	90,013	1	13,654.02	90,012.77
*sodA1*: superoxide dismutase, Mn	GBAA4499	2,665.50	0.00	257,357	1	*2,665.50	257,357.45
RNA polymerase sigma factor *sigA*	GBAA4515	201.86	45.67	88,517	29,661	4.42	2.98
maoC like domain protein	GBAA4836	41.68	22.77	8,751	4,565	1.83	1.92

1 Only data for Control sample listed here – all data included in Supplemental Materials.

2 ssRNAseq measurements are “gene scores” (average hits per nucleotide).

3 nanoString data listed in arbitrary fluorescent units (background subtracted – average of 3 biological replicates).

4 Spearman Rank Correlations (17 df): rho = 0.946 (Control); 0.949 (Cold); 0.947 (EtOH); and 0.949 (NaCl). All p<1E-09.

* Note that gene GBAA4499 had AS signals of 0.00, and so the ratio is listed as the Sense score only (i.e., a denominator of 1, as in nanoString assay – thus, these ratios are an underestimate).

### Sense and AS Transcript Abundance Follows Leading/lagging Strand Chromosome Architecture

**Figure 3:**
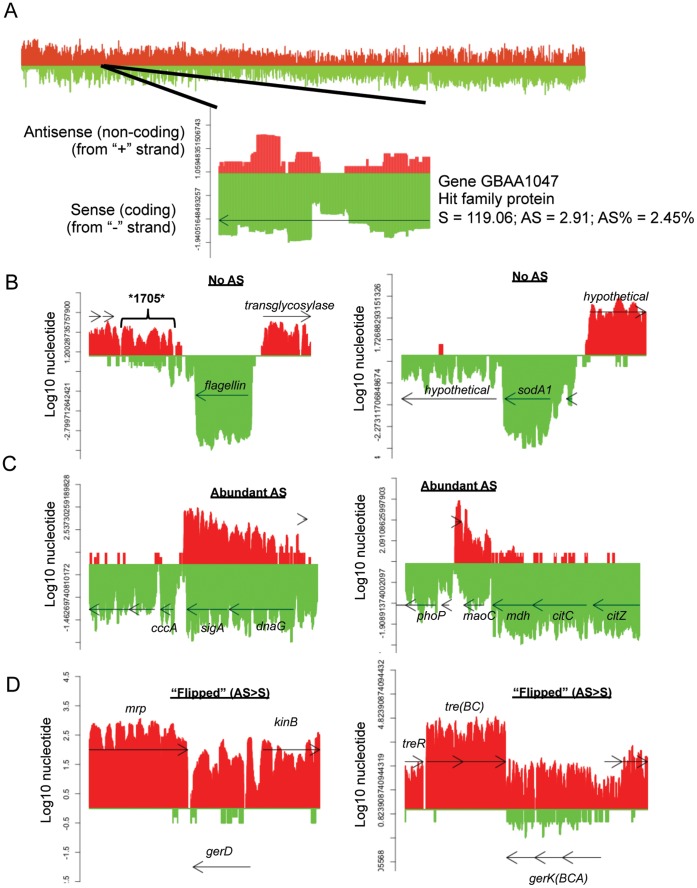
Examples of expression coverage illustrating single nucleotide scores with transcriptional activity of surrounding genomic regions. (A) *B. anthracis* chromosome, from coordinates 1–5.2 million. Red = plus strand signal; Green = minus strand signal. *For all plots, y-axis = log10 of the average coverage of each nucleotide*. *(i.e., how many times each nucleotide was counted per RNA-seq sequencing run – normalized by total coverage)* for 4 biological replicates of Control bacteria (grown in rich broth). Detail inset for (A) is locus tag GBAA1047, coordinates 1033577 to 1034011. (B-D) Expression coverage plots illustrating 3 categories of sense plus AS transcription for 6 genomic regions. Genome Coordinates from *B. anthracis* Ames Ancestor genome: (B) left = 1605215-1608898 (GBAA1703-07); right = 4092484-4095786 (GBAA4498-4500) (C) left = 4105611-4111707 (GBAA4512-18); right = 4397107-4402721 (GBAA4833-39) (D) left = 141515-144006 (GBAA0147-79); right = 646019-655528 (GBAA0630-37). NOTE: See Table S3 for average gene scores with Standard Deviations and 95% Confidence Intervals for 4 biological replicates. *e.g.,* flagellin in panel (B) has a square root transformed average and SD of: Sense = 85.62±0.25 and AS = 0.00; *sigA* in panel (C) is Sense = 14.06±2.38 and AS = 6.59±1.75; and, *gerD* in panel (D) is Sense = 0.46±0.12 and AS = 5.10±1.58.

**Figure 4:**
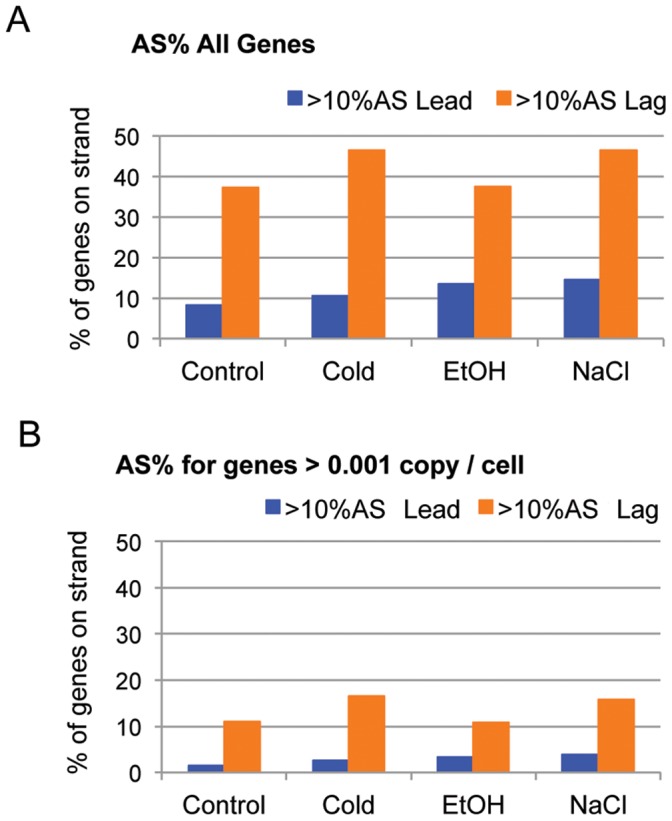
Bar charts illustrating proportions of Antisense percentage scores (AS%) per leading and lagging strands. (A) Percentages of genes on leading and lagging strands with greater than 10% AS scores. Percentages calculated per all annotated genes on strands (Leading = 4,049 genes; Lagging = 1458 genes). (B) Same as A, except only considering genes with sense-directed transcription of>2.5, representing those genes present at approximately 0.001 copy per cell.

**Figure 5:**
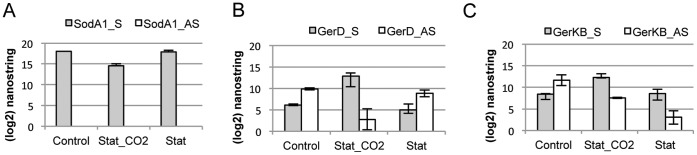
Example of sense to AS ratio flip for two germination proteins as measured by nanoString. Bar charts illustrating Sense (“S”) and Antisense (“AS”) nanoString nCounter scores for: (A) Very high expression of *sodA1* gene (GBAA4499) in 3 unique RNA pools, with 0.00 AS activity in all samples; (B) and (C) changes in Sense to Antisense levels in two germination proteins (*gerD* and *gerKB*, respectively), from exponential to stationary phase with CO2 and without CO2. Error bars = standard deviations of 3 biological replicates for Control and Stationary + CO2, and 2 biological replicates for Stationary phase.

**Figure 6:**
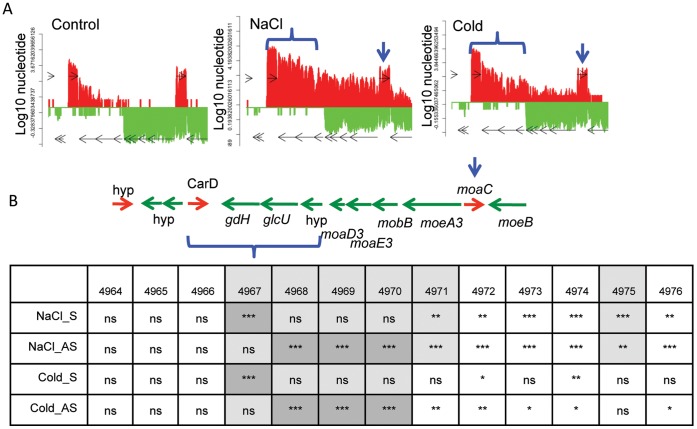
Coverage plots comparing one gene region in three different growth conditions with gene expression analysis. (A) Single nucleotide coverage plots for: Control (left), osmotic stress (center – NaCl) and cold stress (right – Cold) for *B. anthracis* genome region coordinates 4508869-4516678 (GBAA4964-4976). hyp = hypothetical protein; *gdH* = glucose-1-dehydrogenase; *glcU* = glucose uptake protein; *moaD3* = molybdopterin converting factor; *moaE3* = molybdopterin synthase subunit; *mobB* = molybdopterin guanine dinucleotide biosynthesis protein; *moeA3* = molybdopterin biosynthesis protein; *moaC* = GTP cyclohydrolase subunit; and *moeB* = molybdopterin biosynthesis protein. y-axis = log_10_ of individual nucleotide score. Traces are plotted at the single nucleotide level from the additive RNA-seq sequence data. (B) Illustration of gene region structure and table showing results of 1-Way ANOVA with Bonferroni multiple comparisons test analysis of gene expression (4 biological replicates for each condition) for both the Sense and AS directions between the Control Sample versus NaCl (top 2 rows) and versus Cold (bottom two rows). Top row is locus tag number. ns = not significant; ***p<0.0001; **p<0.01; *p<0.05. (Gray shaded areas represent genes that were differentially expressed in an Affymetrix microarray experiment - Passalacqua & Bergman, unpublished).

It is widely accepted that genes are distributed throughout bacterial chromosomes in a non-random way (for an excellent review, see [32]). For instance, in most bacterial replicons, the majority of genes are present on the leading strands [32], [33], [34], which is putatively favorable because collisions between RNA polymerases performing transcription and DNA polymerases performing replication are less likely if both enzymes proceed in the same direction. Additionally, because chromosome replication in bacteria proceeds from a single origin, the genes near that origin are often present in the cell at a higher copy number than the rest of the chromosome, leading to a potential gene dosage effect [32] caused by the presence of more physical copies of the DNA synthesized nearer to the origin than farther away from it (Figure 2A). Given these facts, we were interested in determining the effects of genome architecture on both sense and AS transcript abundance by analyzing gene expression by gene locale. Specifically, we looked at (i) the genome-wide distributions and abundance of the sense and AS signals separately, both genome-wide and per the leading and lagging strands (Figure 2B–E); and then, (ii) explored the combined sense-plus-AS signals of each protein-coding gene (Figures 3–6) (next section). Figure 2A diagrams the ∼5.2 Mb *B. anthracis* chromosome, showing the number of genes located on the leading and lagging strands, and also illustrating four approximate quadrants that divide the genes into their relative proximity to the origin of replication.

We performed a non-parametric Mann-Whitney test of the normalized square-root transformed gene scores between leading and lagging strands (4 biological replicates for each of 4 conditions). The null hypothesis for this analysis was that all genes, regardless of chromosomal strand locale, have an equal chance of being transcribed to any level, in either the sense or AS direction, and that transcription levels in either the sense or AS direction are evenly dispersed across the entire chromosome, regardless of strandedness or proximity to the origin. The results showed a significant difference in medians when comparing the leading versus lagging strands both for Sense and Antisense data (p.<0.0001); however, the trends displayed a consistent directionality that can better be observed in the descriptive statistics in Table 2 and in the score distributions in Figures 2B-C. For all experiments, the overall trends showed that the genome-wide expression medians were (1) decreased on the lagging strand in the sense direction; and (2) increased on the lagging strand in the AS direction. Table 2 lists the descriptive statistics including medians, 75th percentiles, maximum gene scores, the means across four biological replicates with standard deviation, and the upper and lower 95% confidence intervals of the means to give an idea of the spread inherent in such large datasets. The ratios of the medians of Leading to Lagging strand expression were>1.2 for sense and<0.25 for AS for all four growth conditions. Figure 2B–C illustrates distributions similarly to those in Figure 1, but instead of showing overall Sense plus Antisense, they illustrate the distribution of leading versus lagging strand signals for either Sense OR Antisense scores as a percentage of genes per strand for a given range of gene expression values (normalized log_2_ hits/nucleotide). For the most part, the abundance of sense-directed transcripts is proportionately distributed on the both the leading and lagging strands for scores between 2^1^ and 2^8^; however, the distribution shows that lagging strand genes tend to be expressed in the sense direction at slightly lower levels, with more leading strand genes having the highest scores. Also note that almost all of the annotated genes, whether on the leading or lagging strand (∼98% leading ∼96% lagging), were transcribed in the sense direction. This is in contrast to the plots in Figures 2C and S3 that illustrate AS score frequencies. Here, a sharp bias toward higher expresion for the AS signals on the lagging strand is apparent for all four samples. Unlike the sense-directed transcripts, we see that many (∼30–40%) of the annotated genes on the leading strand have an AS score of 0.00, whereas relatively few genes on the lagging strand (∼10–12%) have no detectable AS transcription. The consistency of these trends across four unique growth conditions suggests that perhaps much of the greater abundance of AS transcription on the lagging strand could be due to stochastic noise generated by the predicted enzyme collisions that occur during RNA transcription and DNA replication when the respective polymerase enzymes are moving in opposite directions [35], [36], with only a subset of AS expression existing as possible overt gene regulation.

**Table 2:** Descriptive statistics of Sense and Antisense gene expression per Leading and Lagging strands.

	Growth	*Sense_Lead	*Sense_Lag	*S_R	*AS_Lead	*AS_Lag	*AS_R
**median**	Control	3.43	2.66	1.3	0.062	0.465	0.13
	Cold	3.26	2.45	1.3	0.092	0.681	0.13
	EtOH	3.72	2.92	1.3	0.125	0.482	0.25
	NaCl	2.73	2.26	1.2	0.110	0.593	0.18
**75% percentile**	Control	7.76	5.53		0.165	0.852	
	Cold	7.31	4.96		0.250	1.193	
	EtOH	8.76	7.05		0.300	0.951	
	NaCl	6.24	4.51		0.300	1.063	
**maximum**	Control	120.5	85.63		41.24	24.14	
	Cold	127	83.02		37.56	23.62	
	EtOH	149.2	98.36		19.55	32.83	
	NaCl	131.0	103.9		54.73	29.05	
**mean (SD)**	Control	6.9 (11.5)	4.5 (6.2)	1.5	0.166 (0.788)	0.730 (1.229)	0.22
	Cold	6.85 (12.2)	4.24 (6.1)	1.6	0.228 (0.77)	0.969 (1.33)	0.23
	EtOH	7.13 (10.0)	5.56 (7.66)	1.3	0.262 (0.644)	0.861 (1.649)	0.30
	NaCl	6.22 (12.1)	4.03 (6.7)	1.5	0.296 (1.18)	0.851 (1.308)	0.35
**(lower;upper) 95% CI**	Control	(6.55; 7.26)	(4.19; 4.833)		(0.142; 0.191)	(0.667; 0.794)	
	Cold	(6.48; 7.23)	(3.92; 4.55)		(0.204; 0.251)	(0.900; 1.038)	
	EtOH	(6.92; 7.44)	(5.16; 5.95)		(0.242; 0.282)	(0.776; 0.946)	
	NaCl	(5.85; 6.60)	(3.69; 4.38)		(0.259; 0.332)	(0.783; 0.918)	

* Square root transformed scores from 4 biological replicates - S_R and AS_R are Leading to Lagging Strand ratios for Sense and Antisense data. Leading strand (n = 4049); Lagging strand (n = 1458).

The higher abundance of lagging strand AS transcription and a possible gene dosage effect can be seen in Figure 2D–E. The graphs illustrate the median gene expression (normalized square root transformed scores) with interquartile ranges for gene expression grouped by their proximity to the origin of replication (per Figure 2A). First, the data show a consistent downward trend in median gene expression as gene locale becomes more distant from the origin (“bottom” genes). This is true for both the leading and lagging strands for sense transcription, but only on the lagging strand for AS transcription. Figure 2E further highlights the paucity of leading strand AS abundance compared to the lagging strand for all samples (see differences in y-axes).

For all four experiments, the genes with raw count scores>900 hits/nucleotide (those estimated to be present at a level of>1 copy per cell [4], considered “highly abundant”) were overrepresented on the leading strand (∼ 3% of total leading strand genes, and ∼1% of total lagging strand genes; chi-square test of proportions p = <0.001). These highly expressed genes also had extremely low raw AS scores ranging from 0.00–0.29. Thus, the most highly expressed genes in all samples had virtually no transcription from the non-coding strand. These very abundant genes included many crucial housekeeping genes (e.g., almost all 50S and 30S ribosomal proteins, elongation factor Tu and EF-2, RNA polymerase subunits, single-stranded binding proteins, etc.), which represented about 35–40% of these genes for all samples except the EtOH condition (∼21%). The chaperones GroEL/GroES, the superoxide dismutase *sodA1*, and the S-layer proteins *sap*, were also in this very highly abundant group for all samples, which has been seen before [4], [20]. One gene of note, however, was the inclusion of the RNA binding protein Hfq (GBAA3842) as being in the>900 category only for the EtOH sample (raw scores ranged from 76–350 for non-EtOH samples), strongly suggesting a role for this protein in this specific type of stress in *B. anthracis*. Lastly, regarding these highly abundant genes, ∼60% of the genes were located in the “top” section of the chromosome, closest to the origin (Fig. 2A), while only 2%–5% of them were located on the “bottom” section, farthest from the chromosome. The only exception was that the EtOH sample had more genes from the “right” and “left” sections (∼50%) than from the “top” (∼45%). Appropriately, a cold shock protein (GBAA2422) located in this “bottom” section (lagging strand) was increased substantially in the Cold sample (>2,000 raw score; 280–670 raw score for non-Cold samples). Conversely in the leading strand “bottom” region, a heat shock protein (GBAA2262) was only expressed to very high levels in EtOH (>2,000 raw score; 8–148 in non-EtOH samples). In general, AS transcription is significantly more likely to be detected from genes on the lagging strand, whereas sense directed transcription on both strands and AS on the lagging strand can be seen to vary in overall levels depending on a gene’s proximity to the origin, with several isolated notable exceptions.

### A Variety of Antisense Patterns

The frequency plots illustrated in Figures 1, 2 and S2 categorize sense and AS scores globally but separately, and so do not convey the balance of sense and AS activity together for individual genes (i.e., whether a gene has both S+AS, only S, only AS, or neither S nor AS). We also had not yet considered transcriptional activity of intergenic regions. Thus, we asked if the patterns of combined sense plus AS transcription for all genes were randomly distributed across individual gene regions, and what the expression profiles looked like when considering surrounding regions. We first calculated a simple AS to sense percentage (AS%) for each gene and looked at the distributions on the leading and lagging strands (Table S3– AS% in the raw score tabs). (Note that the percentages were calculated using the raw, normalized scores as opposed to the square root transformed numbers, since the latter calculation would likely exaggerate the trend). We then considered transcriptional patterns of various classes of genes and visually inspected transcriptional patterns within several genomic regions of interest. Also, because the digital nature of RNA-seq allows intrasample quantification of gene expression levels, we calculated an estimated range of scores that could be expected for transcripts present at a certain copy per cell in the coding direction. The Poisson Cumulative Distribution Function (described previously in [4]) was used to calculate a 95% confidence interval for a range of scores where we could expect a gene to be present at approximately 1, 0.1, 0.01, and 0.001 copies per cell. These confidence intervals were then used as a rough estimation of transcript abundance based on score within 1 growth condition.

For example, Figure 3A is a representative plot showing strand-specific RNA-seq coverage across the entire *B. anthracis* chromosome (one sample - normal growth in rich medium), and it highlights one example of a gene that exhibited both sense and AS transcription. Note that the y-axis represents the log_10_ of the average number of times that each nucleotide was counted as being present in an RNA present under a specific growth condition (i.e., nucleotide coverage). This gene, GBAA1047 (Hit family protein), was transcribed in the sense direction with a raw score of ∼119, suggesting that this transcript is present at ∼0.1 copy per cell. AS signals were also mapped across most of the opposite strand of this gene, resulting in an overall AS score of 2.91 reads per nucleotide, resulting in an AS percentage of 2.45%.

It is generally believed that AS RNA in a cell will bind efficiently to sense copies of its corresponding mRNA, decreasing its ability to act as a template for translation and perhaps lowering its stability as well [11], [12], [37], [38]. However, it is not yet clear how much AS transcription is required to effectively change a given mRNA’s stability or translation, and so it is impossible to claim a percentage of AS transcription that is biologically significant. With this in mind, we chose to examine distributions of genes with>10% and>33% AS%. Figures 4A and S3A show that most of the genes with>10% and>33% AS% are located on the lagging strand (Chi-square test, 1 df, p<0.001), (∼30–40% lagging strand; ∼8–14% leading strand), further supporting the previous combined transcript analysis.

Because many of the genes in this subset seemed to be transcribed at very low levels from both strands (<<0.001 copy per cell), we thought it might be possible that very low-level expression was exaggerating the trend. Also, it is possible that unexpressed and potentially misannotated genes could confound the analysis. It should be noted, however, that a recent study of these RNA-seq data combined with a new protein prediction algorithm suggested that the current *B. anthracis* genome annotation probably has more false negatives than false positives [25]; i.e, is more likely to be missing genes than to contain genes that are wrongly annotated, and remains a high-quality genome annotation. Also, recall that>98% of genes were detected as having sense-directed transcription.

We therefore looked at the AS% distribution using only those genes that were present in the transcriptome at>0.001 copy per cell (raw normalized sense gene score>2.5; approximately 4,000 genes for all 4 conditions), which resulted in the same trend as when all genes were considered (Figures 4B and S3B). Of the genes in this subset, 11–16% of the lagging strand genes had AS%>10%, whereas only 1.5–3.8% of leading strand genes did. Approximately 4–7% of expressed leading strand genes had AS%>33%, while 6–11% of expressed lagging strand genes had an AS% this high.

Because gene expression has been traditionally viewed only from a sense-strand perspective, we created a novel nomenclature for RNA-seq gene expression that encompasses the complex potential of sense plus antisense expression as follows (Table 3): (i) No expression (0.00 both strands); (ii) AS expression only; (iii) Sense expression only; (iv) Low AS (AS%< = 1%); (v) Mid AS (1Sense).

**Table 3:** Categories of Sense + AS expression – numbers of genes per category (n = 5,507 genes).

	*Control	*Cold	*EtOH	*NaCl
No expression (0.00/0.00)	29 (25+4)	26 (25+1)	34 (29+5)	40 (33+7)
No Sense w/AS (0.00/AS+)	55 (17+38)	59 (11+48)	53 (22+31)	63 (18+45)
Sense w/no AS (S+/0.00)	1946 (1772+174)	1721 (1578+143)	1491 (1325+166)	1604 (1451+153)
Low AS% (< = 1%)	1698 (1330+368)	1544 (1299+245)	1760 (1381+379)	1421 (1180+241)
Mid AS% (1	951 (583+368)	1106 (715+391)	1134 (767+367)	1174 (794+380)
High AS% (10	558 (260+298)	722 (335+387)	736 (433+303)	848 (450+398)
Flip (AS>S)	270 (62+208)	329 (86+243)	299 (92+207)	357 (123+234)

* Numbers represent counts of genes in each category per the entire annotated genome. Top number is total. Numbers in parentheses are leading (left) plus lagging (right) strand counts.

Figure 3B–D illustrates the variety of AS patterns that were identified with RNA-seq including transcriptional activity from the surrounding region (several of which were confirmed independently with NanoString technology assays (Table S5)). Figure 3B shows expression plots for two very highly expressed genes, the flagellin gene (left) and the *sodA1* gene (right), both of which displayed a very low or non-existent AS signal within the genes themselves and in the surrounding genes (all plots from Control sample – average transformed nucleotide score for all 4 biological replicates). Note that in the flagellin region, a gene that had been originally annotated, but subsequently removed, appears to be an actively transcribed region (former GBAA1705) – a phenomenon that has been seen before [4], [25]. The high and clean expression of *sodA1* was confirmed by NanoString assay (Tables S4 and S5), and overall, this pattern illustrates a ‘traditional’ vision of “gene expression”, where no transcription takes place from in the AS direction.

Conversely, gene regions pictured in Figure 3C show an extremely unexpected pattern, where AS transcription is almost equal to the sense transcription. For the *sigA;dnaG* genes, this does not appear to be run-on transcription from the opposite strand; rather, it appears as if both strands are being specifically transcribed. This is particularly interesting, since *sigA* is the gene for the putative housekeeping RNA polymerase of *Bacillus* species [39], and it seems counterintuitive that there would be an abundant AS species that may destabilize or inhibit the translation of the *sigA* mRNA. The plot on the right side of Figure 3C shows that the *maoC* gene has a very high AS signal that may be run-on from transcription of a hypothetical gene being expressed on the opposite strand. Note however that the *mdh/citC/citZ* genes, being expressed upstream of *maoC* on the same strand, are not only being transcribed at higher levels than *maoC*, but the AS signals for these genes are very low. The dual-stranded transcription was confirmed with the alternative NanoString assay for *maoC* which detected high signals for both strands of these genes, where the AS signal was slightly lower than the sense signal (Tables S4 and S5).

Lastly, an unusual group of genes had higher expression from the AS strand than in the sense direction. We referred to these genes as “flipped”, and each of the samples had almost 300 genes in this category (5–7% of all genes). Overall, ∼39% of the genes expressed in this category were hypothetical genes, and also included various transporters, enzymes, and putative regulatory proteins. Notably, we observed that two identical groups of proteins were “flipped” in all four growth environments. First, 9 prophage lambda and phage related genes representing at least 3 of 4 prophage lambda regions on the *B. anthracis* genome were flipped in all 4 samples. More notably, we noticed that many of the “flipped” genes were associated with sporulation, germination or spore structure (∼6–7% of “flipped” genes for all four growth conditions). The endospore is a unique physiological aspect of bacterial genera such as *Bacillus*, *Clostridium,* and several others, where the cells are able to form dormant endospores that are resistant to multiple environmental insults. The “flipped” genes included several coding for germination proteins (proteins located in the dormant spore, involved in sensing the environment to exit dormancy), stage V sporulation proteins, and putative spore coat proteins (structural and uncharacterized proteins loaded in the proteinaceous coat layer of the endospore). Notably, almost all of the “flipped” spore-related genes were expressed equivalently in all four growth conditions, suggesting that even under the stresses studied here, these genes display unusual transcription patterns. The *gerD* gene and the *gerKCBA* operon illustrated in Figure 3D represent two such spore-related loci and transcription in the sense direction for these genes was very low, while AS transcription from the opposite strand was quite high. Qualitatively viewing the plots, it seems unlikely that the AS signals here are simply due to run-on from the opposite strand, since the AS transcription within the surrounding genes seems to be distinct from the *gerD* and *gerK* AS signals. Also, we observed that the AS signal for *gerK* was greatly reduced in the EtOH sample, suggesting that this stress condition may be modulating expression of this operon (Table S5). We therefore confirmed the levels of sense and AS abundance independently with the NanoString assay for 7 different germination proteins, as well as a for the spore coat protein, *cotS* (Tables S4 and S5).

If the “flipped” AS signals in the cases of *gerD* and *gerK* represent a regulatory mechanism, it would not be surprising to see this mode of regulation during exponential, aerobic growth, since changes in expression of spore-related proteins are generally observed after stationary phase has commenced [23], and vegetative cells are generally lacking in germination proteins [40]. However, sporulation and germination in *B. anthracis* most likely involves a unique repertoire of proteins from *B. subtilis*, which has been characterized extensively [41], [42] [43], and so it is difficult to truly ascertain if this group of genes is truly overrepresented in the “flipped” category for this particular species.

A previous transcriptome study of *B. anthracis* that included stationary phase transcription (CO_2_ and aerobic) was not strand specific [20], and could not differentiate between sense and AS expression. Therefore, we used the NanoString assay to measure the sense versus AS signals in RNA harvested from those previous studies that interrogated stationary phase expression in CO_2_ and aerobic conditions. Interestingly, in CO_2_-stationary phase, a reversal in AS to sense signal for *gerD* was observed. Additionally, the sense-directed transcription for *gerKB* was increased and inverted for both CO_2_ and aerobic stationary phases (Table S5(V)). Figure 5 illustrates this reversal (NanoString Sense and AS scores), and contrasts the high and clean sense expression of the *sodA1* gene to the mixed and varied sense to AS signals in stationary phase and exponential growth for the *ger* genes. Because stationary phase and growth in CO_2_ are very different from aerobic exponential growth, we might expect to see a large difference in gene expression for some genes between these growth states, and the NanoString data confirmed this. The total reversal of sense to AS signal for several germinant receptor genes may represent a unique level of regulation for these genes, and is currently being investigated further.

Although the samples discussed here were taken from unique growth conditions, stress response-associated changes in gene expression were not the focus of this study, and many excellent studies in this regard for *Bacillus* have been done [20], [44], [45], [46], [47]. However, it should be noted that traditional gene expression microarrays, which are not strand-specific, cannot detect changes in sense to AS signals, so we wondered if changes in AS signals might have occurred between stress conditions. In the NaCl sample, a distinct rise in AS signal was observed for genes GBAA4968-4971. Figure 6 shows expression coverage plots for these genes and their surrounding region for the Control, NaCl and Cold samples. For both the Cold and NaCl samples, *gdH, glcU* and a hypothetical gene (GBAA4968-4970) had a sharp rises in AS signal compared to Control. However, only the NaCl sample had a concomitant steep rise in AS signal for additional adjacent genes, (GBAA4971-74), which are putatively involved in molybdenum metabolism. It is possible that the extension of the AS signal through the *gdH/glcU* region from the CarD gene may be involved in regulating the molybdenum biosynthetic genes, or perhaps represent novel genes located on the opposite strand of the *gdH/glcU* region. The rise in AS signal for the *gdH* gene in NaCl and Cold was confirmed with the NanoString assay (Table S5). More interestingly, we observed that the CarD and *gdh/glcU*/hyp genes were detected as being significantly upregulated in a traditional Affymetrix microarray study using the same RNA samples (Fig. 6B) (Passalacqua & Bergman, *unpublished*). This is important because the rise in signal for the *gdh/glcU*/hyp genes is from the antisense strand only, and this cannot be observed via non-strand specific microarrays, where any rise in transcript signal is assumed to be from the Sense strand. More specifically, the increase in gene expression observed in the microarray experiment could only be misinterpreted as a rise in canonical gene expression, further highlighting the power of strand-specific measurement. This unusual example of not only sense versus AS transcription for individual genes, but of relative amounts of sense and AS activity in a region as a whole, shows that our current paradigms for understanding gene regulation at the transcriptional level are far from complete.

## Discussion

The RNA-seq method has greatly changed the way in which transcriptomes can be viewed and analyzed. In this study, we used a strand-specific version of this method to analyze the *B. anthracis* transcriptome under four different growth conditions, and we uncovered genome-wide patterns of sense and antisense transcription that appear to be driven in part by chromosome architecture, as well as clues suggesting that antisense transcription may play an important role in regulating bacterial gene expression in this Firmicute.

The process of transcription has been shown to be inherently “noisy”, where stochastic effects are a major driving force in gene expression [48], [49], [50]. Several studies have suggested that “synchronized” bacterial populations must be heterogeneous in terms of gene expression (i.e., with different cells expressing different transcriptional repertoires), and also that genes are not simply ‘on’ or ‘off’, but instead are regulated along a continuum of transcriptional activity [51] and can also be spatially oriented [52]. Strand specific RNA-seq has provided the first views of gene expression at a level of detail sufficient to define these phenomena globally.

Widespread transcription of RNA other than that limited to protein-coding regions in eukaryotes is an active field of research, and these still mysterious noncoding RNAs have been referred to as “RNA Dust” [53]. However, a more recent analysis has suggested that the distribution of these “Dark Matter” transcripts is not as widespread in eukaryotes as is currently believed [54], but that they are instead mostly associated with known protein-coding regions. Our analysis of *B. anthracis* transcription seems to reveal patterns that agree with both of these views, where AS RNA signals are overall much lower than sense RNA signals, showing that transcription is predominantly dedicated to making “normal” mRNA; but that AS RNA is generated more frequently from the lagging strand, potentially suggesting that much AS activity is noise from polymerase enzyme conflicts [35]. AS activity also appears in regular and putatively regulated patterns across samples and growth conditions, such as the consistent high AS in an important sigma factor, and the unexpected “flip” in AS to S signals for several germination proteins. Overall, it is clear that rather than there being only one category of antisense transcription, there appears to be a wide variety of AS expression patterns, likely due to multiple mechanisms which are only beginning to be examined [10]. Whereas the seeming bidirectional transcription of the *sigA* region was an example of a consistent AS pattern, many genes with AS activity had more diffuse and “noisy” AS activity. The strong dominance of AS RNA from the lagging strand may be an inherent feature of the phylum of Firmicute bacteria, since Firmicute genomes have very high frequencies of genes on the leading strand [24]. Differentiating between the various AS patterns on a global scale will be a formidable bioinformatic challenge, and teasing out the biological significance of these patterns will likely provide a larger niche in molecular biology studies than was previously thought. Finally, we note that the single stranded RNA-Seq and NanoString nCounter approaches allowed us to view transcription in bacteria from a very unique perspective, and provided a view of phenomena such as the “flipped” transcription observed in the *gerD* and *gerK* regions that would have been difficult or impossible to obtain, or were likely previously misinterpreted, with non-strand specific techniques such as microarrays or qRT-PCR.

It will be interesting to compare the global AS patterns of various bacterial species and genera and ask how AS activity has evolved in different genomes. As our ability to define transcriptomes expands and intensifies, it is clear that our definitions of “gene expression” will need to encompass much more than varying levels of coding strand mRNA levels.

## Materials and Methods

### Bacterial Growth and RNA Isolation

*Bacillus anthracis* strain Sterne 34F_2_ was used for all experiments. For RNA collection, Sterne liquid cultures in LB medium were begun in the morning from an isolated colony grown on TSA-5% sheep’s blood agar plates. All experiments were done with four biological replicates in parallel at 37°C. Baseline transcriptional control cultures (50 ml) (Control) were harvested at OD_600_ 0.4–0.5 after addition of 50 ml of pre-warmed LB medium, and shaking for 10 minutes. For Ethanol (EtOH) stress induction, 50 ml cultures were grown to OD_600_ 0.4–0.5 and 50 ml of LB+12% EtOH were added to the culture, resulting in a final concentration of 6% EtOH. Cells were allowed to grow with shaking for 10 minutes and immediately harvested for RNA. For osmotic stress induction, culture were grown as before, but 50 ml of 1.5 M NaCl was added to the cultures, resulting in a final concentration of 0.75 M NaCl and grown for 10 minutes with shaking. For Cold stress induction, cultures were grown as before, and at OD_600_ 0.4–0.5, 50 ml of previously cooled LB medium (∼4°C) was added to cultures, resulting in an average liquid temperature of 19–20°C. Cultures were then shaken in a refrigerated incubator at 17°C for 10 minutes, and RNA was harvested. Phenol-chloroform extraction, as described previously [4], was used to isolate total RNA, which was enriched using the Ambion MicrobExpress kit. All RNA samples were tested for integrity on a BioRad Experion capillary electrophoresis system. Possible residual DNA was removed by addition of Ambion Turbo DNase.

### Library Preparation and Shotgun Sequencing

Library preparation for Applied Biosystems SOLiD sequencing was done according to the manufacturer’s Whole Transcriptome Library Preparation (see website). Shotgun sequencing (50 bp reads) on an Applied Biosystems SOLiD 3 sequencer was performed at EdgeBio (http://www.edgebio.com/).

### Bioinformatic Analysis

All statistical and quantitative analyses were done on desktop Macintosh computers running OSX in Excel or Prism 5.0 and also using ad hoc perl and R scripts. Raw color space data from SOLiD sequencing was mapped to the *Bacillus anthracis* Ames Ancestor genome (NC007530) [26] using SOCs software [4] with a mismatch cutoff of 5 nucleotides.

Mapping simulation to test for mapping accuracy was done as follows: A simulator was written using perl, whereby input equals number of errors (e) and number of permutations (p) for generating 50 base pair reads begun at each nucleotide of a given genome (*B. anthracis* Ames Ancestor), and output being the number of reads mapped incorrectly. For all p reads from each nucleotide position, a number of errors ranging from 0 to e were generated randomly. The number of reads for a genome of n nucleotides was n-50*p, where p = 200 and e = 6. These reads were then used as input for the SOCs mapping software [29]. Output from SOCs mapping was used to count the number of reads that were correctly and incorrectly mapped with and without errors.

The Poisson distribution function was used to calculate 95% confidence intervals of average gene scores (hits/nucleotide of annotated open reading frame) as described in Supplemental Information previously [4]. Coverage plots were created using custom scripts in R. Analyses were done in Microscoft Excel and Prism 5 (Mann-Whitney Tests and 1-Way ANOVA) for Mac OSX and R (Correlation). Frequency Distributions were created using the average of the replicate log_2_ transformed gene scores for each growth condition and the plots were generated in Microsoft Excel. The Mann-Whitney test comparing gene expression between leading and lagging strands was performed in Prism 5 using the square-root transformed gene expression scores for all 4 replicates for each growing condition (e.g., Control Sense-Leading versus Sense-Lagging; Control Antisense-Leading versus Antisense-Lagging, etc.).

### Nanostring nCounter Gene Expression Analysis

To confirm relative sense to antisense (S/AS) transcript presence in total RNA pools, the nanoString nCounter technology was used (http://www.nanostring.com/) [30], [31]. The same RNA samples that were used for SOLiD sequencing described above were sent to Nanostring at a concentration of 50 ng/µl. Additionally, 3 RNA samples from previous RNA-seq experiments were assayed (Stationary phase in aerobic and CO_2_ growth). Briefly, 50 base pair strand-specific probes were designed at nanoString against 17 unique genes to specifically detect the presence of Sense and/or Antisense RNA. Two sets of probes were designed for each gene: (i) Sense probes; and (ii) Antisense probes. Each probe set was run as a separate experiment where the nanoString probes were hybridized to the sample RNA, and a fluorescence reading represents the output. A housekeeping gene that was shown by RNA-seq to have low antisense transcription and similar levels of sense transcription in each sample was chosen as a positive and normalization control to run in each experiment with only a Sense probe. Each gene was assayed using RNA from 3 separate biological samples. Two genes were used as technical replicates, whereby the same RNA sample was run in triplicate. NanoString runs a titrated spiked control quantitative probe set in each well for each experiment to normalize signal variance. Also, a set of negative probes were added to each well to detect background fluorescence, and the average background fluorescence plus three standard deviations were subtracted from the experimental data to remove background signal. After background subtraction, fluorescence signals were averaged for each gene, resulting in a sense and an antisense average signal.

### Genome Sequences

The raw sequence data and mapped reads have been submitted to the National Center for Biotechnology Information Gene Expression Omnibus (GEO) database under accession number GSE36506.

## Supporting Information

Figure S1 **Frequency distributions for Sense and Antisense signals in 4 transcriptome samples for the** ***B. anthracis*** **pXO1 plasmid.** Plots represent the numbers of genes in each range of scores for both Sense and Antisense signals (x-axis = log_2_ of scores; y-axis = number of genes within each range). Control = exponential growth in rich medium; Cold = 10 minutes at 17°C; EtOH = 10 minutes at 6% Ethanol; and NaCl = 10 minutes in 0.7 M sodium chloride. 0* = score of 0.00.
(TIF)

Figure S2 **Frequency distributions for range of Sense and Antisense scores by leading and lagging strands for Cold, EtOH and NaCl samples.** Frequencies are plotted as percentage of genes on strand (y-axis) per range of Sense (left) or Antisense (right) scores (x-axis = log_2_ scores). Plots use proportions of genes per strand to account for the fact that the leading strand has more genes (higher gene density). 0* = score of 0.00.
(TIF)

Figure S3 **Bar charts illustrating proportions of Antisense percentage scores (AS%) greater than 33% per leading and lagging strands.** (A) Percentages of genes on leading and lagging strands with greater than 33% AS signal. Percentages are per all annotated genes on strands. (B) Same as A, except only considering genes with Sense transcriptional scores>2.5, representing those genes present at approximately 0.001 copy per cell (i.e., genes considered to be “on”).
(TIF)

Table S1 SOLiD single-stranded RNA-seq Sequencing Coverage.
(DOCX)

Table S2 Correlation analysis (Spearman Rank Correlations) of 16 single stranded RNA-seq experiments (single nucleotide correlation for chromosome and pXO1 plasmid, including plus and minus strands).
(XLSX)

Table S3 RNA-seq gene expression analysis for 16 samples. Includes normalized Raw and Square Root transformed data for 4 different growth conditions.
(XLSX)

Table S4 Side-by-side strand-specific expression measurements of 17 genes by both RNA-seq and nanoString nCounter Technology for Control Sample with Sense to Antisense Ratios.
(DOCX)

Table S5 nanoString nCounter data and analyses.
(XLSX)
