# Supplementary figures and images for: Strand-Specific RNA-Seq Reveals Ordered Patterns of Sense and Antisense Transcription in Bacillus anthracis
Source: PLoS One. 2012 Aug 22;7(8):e43350. doi: 10.1371/journal.pone.0043350 (PMC3425587; doi:10.1371/journal.pone.0043350)

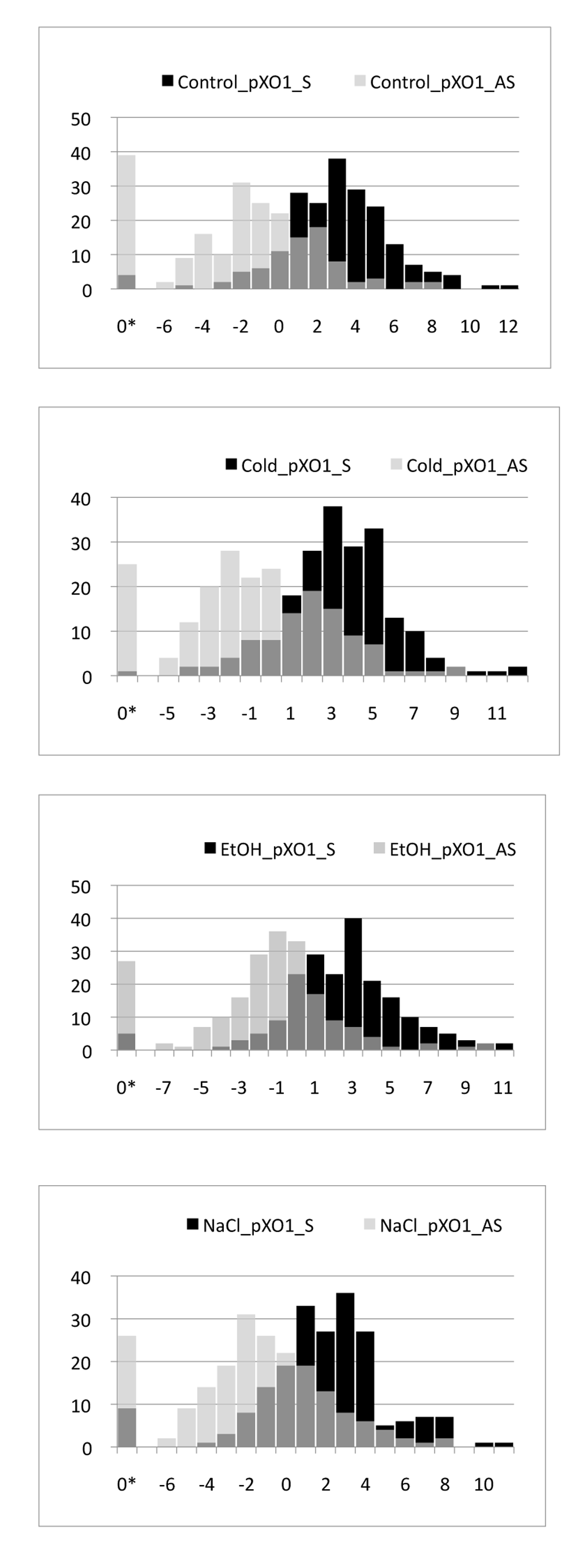

Supplement: Figure S1 — Frequency distributions for Sense and Antisense signals in 4 transcriptome samples for the B. anthracis pXO1 plasmid. Plots represent the numbers of genes in each range of scores for both Sense and Antisense signals (x-axis = log2 of scores; y-axis = number of genes within each range). Control = exponential growth in rich medium; Cold = 10 minutes at 17°C; EtOH = 10 minutes at 6% Ethanol; and NaCl = 10 minutes in 0.7 M sodium chloride. 0* = score of 0.00. (TIF) [file pone.0043350.s001.tif]

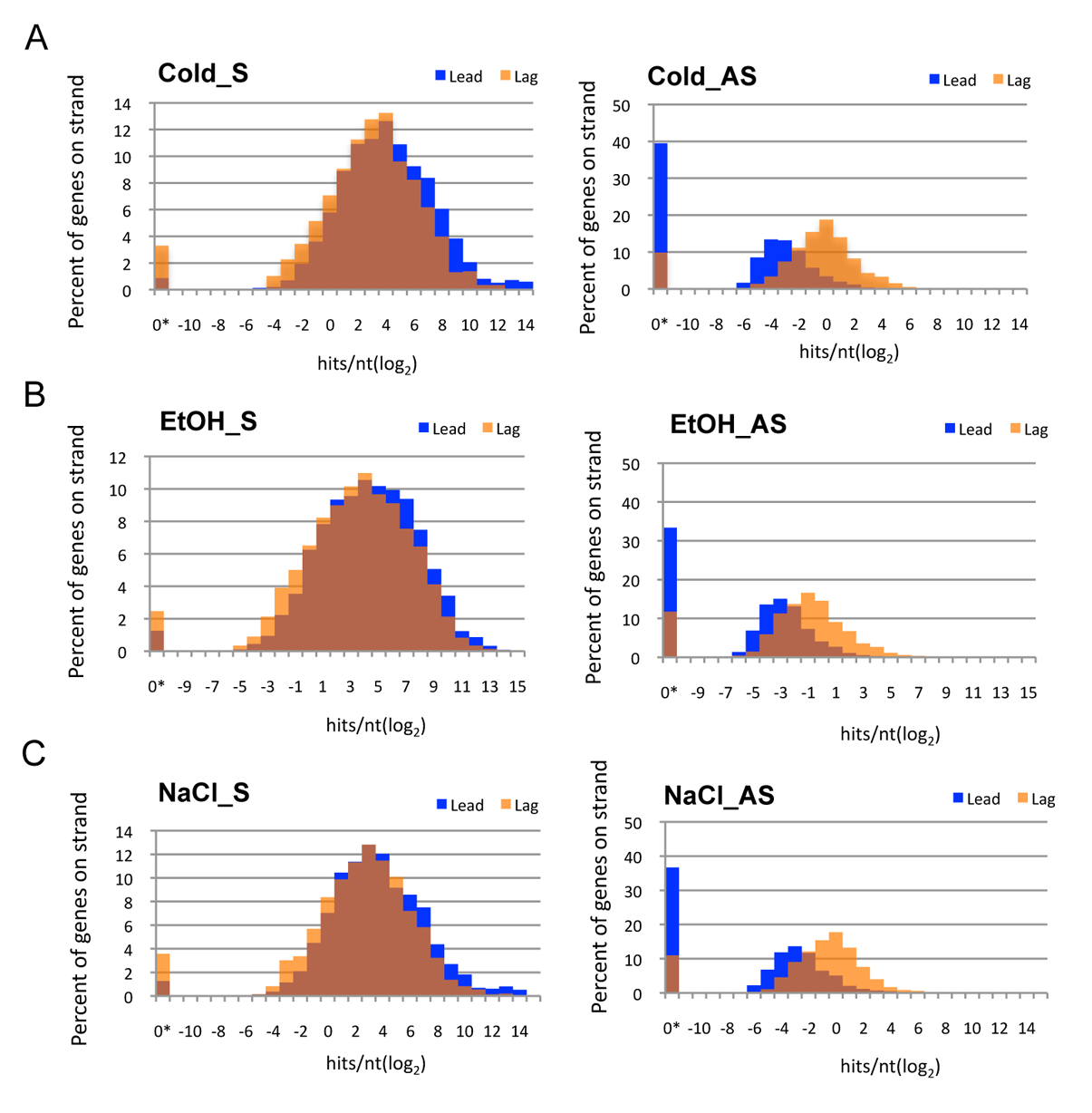

Supplement: Figure S2 — Frequency distributions for range of Sense and Antisense scores by leading and lagging strands for Cold, EtOH and NaCl samples. Frequencies are plotted as percentage of genes on strand (y-axis) per range of Sense (left) or Antisense (right) scores (x-axis = log2 scores). Plots use proportions of genes per strand to account for the fact that the leading strand has more genes (higher gene density). 0* = score of 0.00. (TIF) [file pone.0043350.s002.tif]

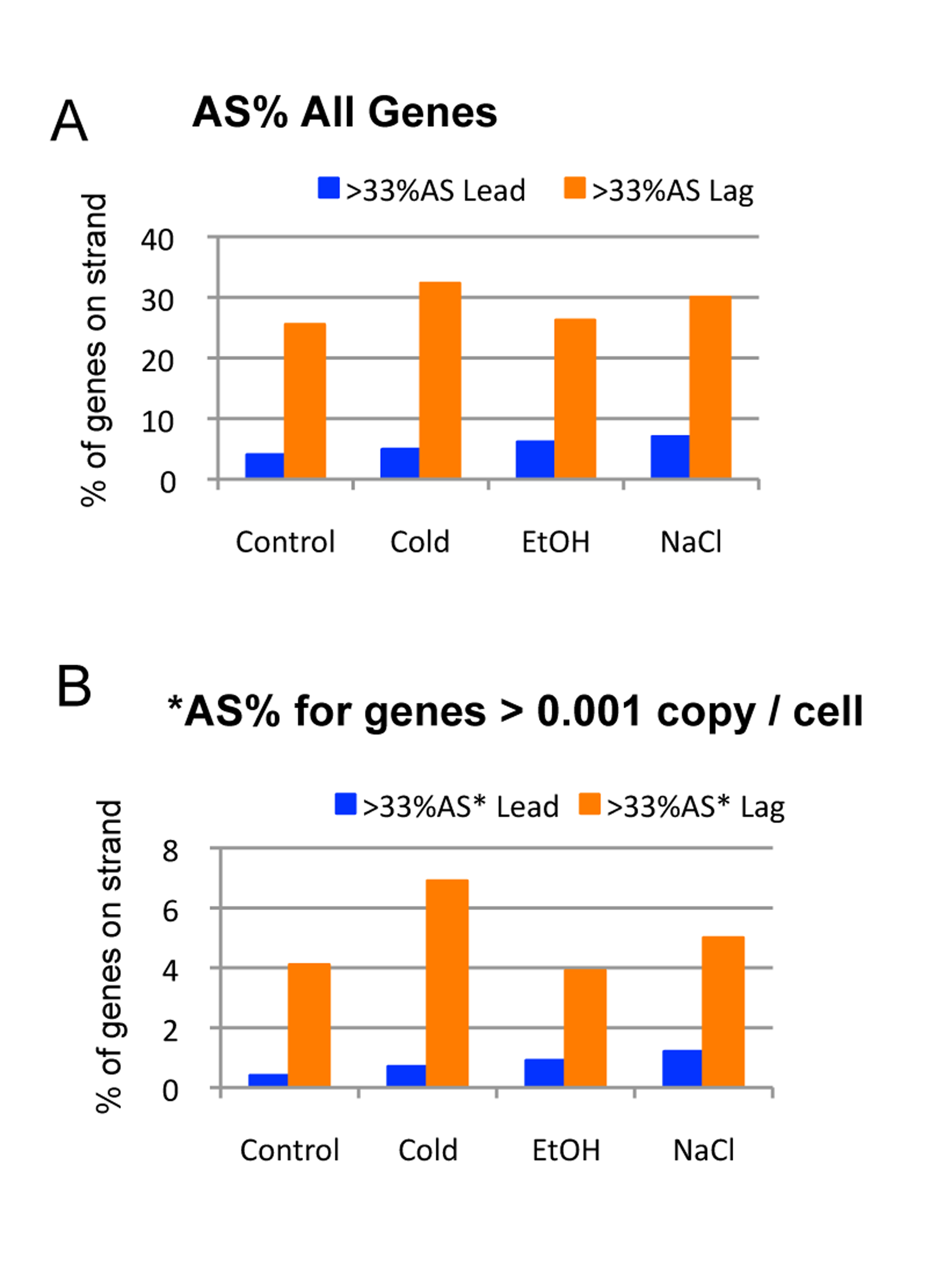

Supplement: Figure S3 — Bar charts illustrating proportions of Antisense percentage scores (AS%) greater than 33% per leading and lagging strands. (A) Percentages of genes on leading and lagging strands with greater than 33% AS signal. Percentages are per all annotated genes on strands. (B) Same as A, except only considering genes with Sense transcriptional scores>2.5, representing those genes present at approximately 0.001 copy per cell (i.e., genes considered to be “on”). (TIF) [file pone.0043350.s003.tif]
