# Supplementary material for: Strand-Specific RNA-Seq Reveals Ordered Patterns of Sense and Antisense Transcription in Bacillus anthracis
Source: PLoS One. 2012 Aug 22;7(8):e43350. doi: 10.1371/journal.pone.0043350 (PMC3425587; doi:10.1371/journal.pone.0043350)
Supplement: Table S1 — SOLiD single-stranded RNA-seq Sequencing Coverage. (DOCX) [file pone.0043350.s004.docx]

Table S1. SOLiD ssRNA-seq Sequencing Coverage

| **Sample** | **Total Reads** | **Reads mapped** | **Unambiguous Reads mapped** | **Unambiguous bases (Mb)** | **Total bases (Mb)** | **Fraction unambiguous** |
| --- | --- | --- | --- | --- | --- | --- |
| Control1 | 36,115,277 | 20,486,781 | 14,366,374 | 718.32 |  | 0.7013 |
| Control2 | 30,039,256 | 15,975,581 | 9,537,385 | 476.87 |  | 0.5970 |
| Control3 | 23,526,394 | 8,314,825 | 4,758,924 | 237.95 |  | 0.5723 |
| Control4 | 27,220,671 | 11,582,423 | 8,017,749 | 400.89 | **1834.02** | 0.6922 |
|  |  |  | **Total = 36,680,432** |  |  |  |
| Cold 1 | 22,020,295 | 9,028,604 | 5,850,459 | 292.52 |  | 0.6480 |
| Cold 2 | 22,901,022 | 13,110,786 | 7,922,654 | 396.13 |  | 0.6043 |
| Cold 3 | 26,460,902 | 12,998,620 | 7,594,071 | 379.70 |  | 0.5842 |
| Cold 4 | 25,341,759 | 13,625,689 | 7,427,666 | 371.38 | **1439.74** | 0.5451 |
|  |  |  | **Total = 28,794,850** |  |  |  |
| EtOH 1 | 18,191,289 | 8,803,146 | 4,942,916 | 247.15 |  | 0.5615 |
| EtOH 2 | 23,007,203 | 11,276,261 | 6,991,382 | 349.57 |  | 0.6200 |
| EtOH 3 | 23,516,435 | 11,799,119 | 6,691,556 | 334.58 |  | 0.5671 |
| EtOH 4 | 30,834,496 | 16,095,666 | 8,009,294 | 400.46 | **1331.76** | 0.4976 |
|  |  |  | **Total = 26,635,148** |  |  |  |
| NaCl 1 | 31,255,068 | 18,172,198 | 8,059,244 | 402.96 |  | 0.4435 |
| NaCl 2 | 49,175,693 | 22,989,806 | 10,156,801 | 507.84 |  | 0.4418 |
| NaCl 3 | 28,294,680 | 12,439,350 | 5,688,459 | 284.42 |  | 0.4573 |
| NaCl 4 | 27,638,072 | 10,934,985 | 4,240,583 | 212.03 | **1407.25** | 0.3878 |
|  |  |  |  |  |  |  |
| **Total reads** | **445,538,512** |  | **Total = 28,145,087** |  | **Total = 6012.78** | **Average = 0.5526** |
| **Total Mb** | **22276.9256** |  |  |  |  |  |
