# Supplementary material for: Strand-Specific RNA-Seq Reveals Ordered Patterns of Sense and Antisense Transcription in Bacillus anthracis
Source: PLoS One. 2012 Aug 22;7(8):e43350. doi: 10.1371/journal.pone.0043350 (PMC3425587; doi:10.1371/journal.pone.0043350)
Supplement: Table S4 — Side-by-side strand-specific expression measurements of 17 genes by both RNA-seq and nanoString nCounter Technology for Control Sample with Sense to Antisense Ratios. (DOCX) [file pone.0043350.s007.docx]

Table S4: Side-by-side strand-specific expression measurements of 17 genes by both ssRNA-seq and nanoString Technology^1^ for the Control Sample with Sense to Antisense Ratios.

| **Gene name** | **locus_tag** | **^2^Sense**  **ssRNAseq** | **^2^AS**  **ssRNAseq** | **^3^Sense**  **nanoString** | **^3^AS**  **nanoString** | **^4^S:AS Ratio**  **ssRNAseq** | **^4^S:AS Ratio**  **nanoString** |
| --- | --- | --- | --- | --- | --- | --- | --- |
| spore germination protein *gerD* | GBAA0148 | 0.22 | 27.91 | 71 | 943 | 0.01 | 0.08 |
| spore germination protein *gerKB* | GBAA0634 | 1.01 | 20.81 | 351 | 3,973 | 0.05 | 0.09 |
| spore germination protein *gerPD* | GBAA1146 | 1.75 | 1.59 | 408 | 77 | 1.10 | 5.28 |
| germination protein *gerN* | GBAA1639 | 92.64 | 0.46 | 18,677 | 115 | 199.75 | 162.04 |
| spore germination protein *gerAA* | GBAA3150 | 14.74 | 0.05 | 4,707 | 2 | 277.54 | 2,078.11 |
| spore germination protein *gerHB* | GBAA4985 | 1.01 | 0.76 | 185 | 194 | 1.34 | 0.96 |
| spore germination protein *gerXA* | GBAA_pXO1_0157 | 12.05 | 0.21 | 1,160 | 6 | 56.51 | 198.14 |
| acid-soluble spore protein H | GBAA0613 | 116.80 | 5.27 | 25,903 | 1,844 | 22.15 | 14.04 |
| 3-deoxy-7-phosphoheptulonate synthase | GBAA2958 | 600.93 | 0.04 | 90,013 | 1 | 13,654.02 | 90,012.77 |
| lpxtg-motif cell wall anchor domain protein | GBAA3254 | 173.08 | 0.03 | 35,599 | 21 | 5,132.26 | 1,666.85 |
| prophage lambdaba02, site-specific recombinase, phage integrase family | GBAA4134 | 36.65 | 9.44 | 11,794 | 763 | 3.88 | 15.46 |
| transcriptional regulator, arsR family | GBAA4483 | 391.01 | 4.10 | 275,733 | 1,161 | 95.45 | 237.54 |
| *sodA1*: superoxide dismutase, Mn | GBAA4499 | 2,665.50 | 0.00 | 257,357 | 1 | *2,665.50 | 257,357.45 |
| RNA polymerase sigma factor *sigA* | GBAA4515 | 201.86 | 45.67 | 88,517 | 29,661 | 4.42 | 2.98 |
| maoC like domain protein | GBAA4836 | 41.68 | 22.77 | 8,751 | 4,565 | 1.83 | 1.92 |
| *gdH*: glucose-1-dehydrogenase | GBAA4968 | 0.43 | 6.58 | 124 | 3,221 | 0.07 | 0.04 |
| spore coat protein cots | GBAA5188 | 0.62 | 32.83 | 236 | 8,251 | 0.02 | 0.03 |
| 4-oxalocrotonate tautomerase | GBAA5626 | 109.80 | 22.57 | 80,309 | 7,207 | 4.87 | 11.14 |
| protective antigen | GBAA_pXO1_0164 | 9.95 | 0.59 | 3,317 | 327 | 16.97 | 10.14 |

^1^Only data for Control sample listed here – all data included in Supplemental Table S5

^2^ssRNAseq measurements are “gene scores” (average hits per nucleotide)

^3^nanoString data listed in arbitrary fluorescent units (background subtracted – average of 3 biological replicates)

^4^Spearman Rank Correlations (17 df): rho = 0.946 (Control); 0.949 (Cold); 0.947 (EtOH); and 0.949 (NaCl). All p < 1E-09.

*Note that gene GBAA4499 had AS signals of 0.00, and so the ratio is listed as the Sense score only (i.e., a denominator of 1, as in nanoString assay – thus, these ratios are an underestimate).
